# Serial Changes in Vitamin D Status in Patients During Severe Acute Respiratory Distress Syndrome and Extracorporeal Membrane Oxygenation

**DOI:** 10.3390/medicina61050901

**Published:** 2025-05-16

**Authors:** Martina Hermann, Jelena Poslussny, Gernot Gerger, Helmuth Haslacher, Georg Mayrhofer, Verena Eva Tretter, Mathias Maleczek, Cem Ekmekcioglu

**Affiliations:** 1 Medical University of Vienna, Department of Anaesthesia, Intensive Care Medicine and Pain Medicine, Clinical Division of General Anaesthesia and Intensive Care Medicine, 1090 Vienna, Austria; 2 Ludwig Boltzmann Institute for Digital Health and Patient Safety, 1090 Vienna, Austria; 3 Medical University of Vienna, Department of Laboratory Medicine, 1090 Vienna, Austria; 4 Medical University of Vienna, Department of Environmental Health, Center for Public Health, 1090 Vienna, Austria

**Keywords:** vitamin D, 25-hydroxyvitamin D, 1,25-dihydroxyvitamin D, acute respiratory distress syndrome, extracorporeal membrane oxygenation, COVID-19, intensive care medicine, critical illness

## Abstract

*Background and Objectives*: Therapeutic interventions, such as extracorporeal membrane oxygenation (ECMO) therapy, in patients suffering from severe acute respiratory distress syndrome (ARDS) may reduce their vitamin D levels. Many observational studies have shown associations between poor outcomes and low vitamin D levels in critically ill patients. This retrospective study primarily aimed to investigate the time-dependent changes in vitamin D levels and the correlation of vitamin D levels with disease severity and inflammatory markers in patients suffering from ARDS receiving ECMO therapy. *Materials and Methods*: This study used a longitudinal approach to assess the serial changes and the correlations of vitamin D levels (25-hydroxyvitamin D (25(OH)D) and 1,25-dihydroxyvitamin D (1,25(OH)2D)) with disease severity and inflammatory markers in 24 invasively mechanically ventilated (IMV) patients treated using ECMO over a period of 17 days. Most of the patients in this study were suffering from severe coronavirus disease 2019 (COVID-19) (*n* = 19; 79%). Serial blood samples collected during routine blood draws were retrospectively analyzed to assess the dynamics of their vitamin D levels over 17 days of ICU therapy. *Results*: Hypovitaminosis D (25(OH)D ≤ 50 nmol/L) was prevalent in 18 (75%) patients, while values of 25(OH)D lower than 30 nmol/L were measured in 5 patients (21%), indicating severe deficiency. Additionally, 1,25(OH)2D showed a significant decrease within the first 11 days of intensive care unit (ICU) treatment (these levels dropped by 28%; *p* = 0.03) and then remained at similar levels throughout the observational period; 25(OH)D levels remained largely unchanged during the observation period. We observed that 25(OH)D showed a significant negative correlation with C-reactive protein (CRP) (*p* = 0.04), and that 25(OH)D and 1,25(OH)2D levels did not show correlations with disease severity. *Conclusions*: Patients suffering from severe COVID-19 ARDS showed a significant decrease in their 1,25(OH)2D levels from day 0 to day 11 in the ICU. Therefore, routine vitamin D substitution and monitoring in critically ill patients, especially for patients suffering from ARDS treated with ECMO, should be carried out to prevent hypovitaminosis D. In addition, vitamin D may be associated with inflammation. Further studies are necessary to elucidate the mechanisms behind these retrospective observations.

## 1. Introduction

Acute respiratory distress syndrome (ARDS) is the most severe form of respiratory failure and has a variety of causes [1]. Since 2020, coronavirus disease 2019 (COVID-19) has become the most frequent reason for ARDS all over the world. Due to its well-documented immunomodulatory properties, vitamin D gained importance during the COVID-19 pandemic [2,3,4], with large amounts of data being published on 25-hydroxyvitamin D (25(OH)D) status, hypovitaminosis D, vitamin D supplementation, and the risk of SARS-CoV-2 infection and severe COVID-19 progression (as summarized by Rust and Ekmekcioglu [5]). In this regard, vitamin D deficiency is strongly associated with intensive care unit (ICU) treatment and worse outcomes in critically ill patients suffering from COVID-19 [5,6,7,8]. A high percentage of critically ill patients admitted to the ICU suffer from low vitamin D levels [9].

In particular, the serum-circulating form of vitamin D—25(OH)D—and its most biologically active form—1,25-dihydroxyvitamin D (1,25(OH)2D)—intricately regulate the antiviral immune system’s response to inflammation and infection [10,11]. Furthermore, the pathogenesis of patients with severe COVID-19 disease admitted to the ICU is characterized by dysregulated immune responses and the overwhelming release of proinflammatory cytokines [12]. Vitamin D has emerged as a modulator of cytokine production and inflammatory pathways, raising speculation about its potential role in attenuating cytokine-storm-associated complications in SARS-CoV-2 patients [13].

However, it has not conclusively been proven whether low vitamin D status is a risk factor for COVID-19 and other inflammatory diseases or whether it is a consequence of the disease. In this context, low levels of vitamin D could be misinterpreted as a risk parameter. Inflammation itself could lower 25(OH)D concentrations or, conversely, vitamin D values within the reference range could reduce inflammation [14]. On the one hand, Duncan et al. [15] described that increasing C-reactive protein (CRP) levels correlate negatively with 25(OH)D [15]. On the other hand, a recently published “umbrella” meta-analysis comprising 23 meta-analyses of 21,148 participants showed that vitamin D supplementation led to a reduction in CRP and Tumor Necrosis Factor (TNF)-alpha [16]. To summarize, the routine measurement and serial monitoring of vitamin D levels should be a fixed part of ICU treatment to provide an adequate vitamin D status [6].

Little is known regarding the short-term serial changes in vitamin D levels in humans with inflammation or disease. For example, changes in postoperative 25(OH)D concentrations were shown within patients after knee arthroplasty [17]. Similarly, significant decreases in 25(OH)D levels one day after total hip arthroplasty surgery [18] or after open heart surgery [19] have been observed.

In addition, few papers have studied the kinetics of vitamin D in critically ill patients [20,21,22], showing unchanged [22] or modified kinetics over a period of a few days [21]. Optimal doses for vitamin D substitution in critically ill patients have not been sufficiently clarified [23]. In summary, recent studies have described that inflammation (especially high CRP values) can influence 25(OH)D status. Also, only a few papers have presented data on the short-term-dependent changes in vitamin D status in critically ill patients. However, to the best of our knowledge, this is the first study investigating serial changes in vitamin D levels including different vitamin D supplementation regimes in critically ill patients suffering from severe (COVID-19- and non-COVID-19-related) ARDS treated with extracorporeal membrane oxygenation (ECMO) therapy. This could be relevant for potential vitamin D supplemental protocols in these patients. Therefore, the aim of this study was the investigation and correlation of serial changes in 25(OH)D and 1,25(OH)2D levels with inflammatory parameters and outcome in this population of patients.

## 2. Materials and Methods

### 2.1. Study Population

Between March 2020 and January 2021, 24 patients admitted to the ICU diagnosed with severe ARDS (COVID-19- and non-COVID-19-related), treated by invasive mechanical ventilation and ECMO therapy, were included in this clinical trial.

### 2.2. Data Collection

Assessed data included baseline demographics and physiological parameters prior to ECMO initiation, as well as clinical and laboratory parameters during the ICU stay. Simplified Acute Physiology Score III (SAPS III) [24] was calculated at ICU admission. Patient identification and data collection were conducted using the patient data management system’s routine documentation (ICCA©, Philips, Amsterdam, The Netherlands).

### 2.3. Blood Samples

Within this study, we performed a retrospective analysis of serial leftover blood samples obtained during routine blood draws to investigate the dynamics of vitamin D levels in patients admitted to the ICU, mainly due to severe COVID-19 ARDS, undergoing ECMO therapy. Notably, 25(OH)D serum levels are not routinely measured in ICU patients. Blood samples (if available) were collected pre-ECMO (day 0) and during ECMO therapy (day 1, day 2, day 3, day 7, and day 14) and stored immediately in the research laboratory or the Medical University Vienna Biobank at the Department of Laboratory Medicine [25]. Routine laboratory parameter analyses, including the routine assessment of vitamin D serum levels, were conducted at the Central Clinical Laboratory using state-of-the-art equipment and standardized assay protocols. Routine laboratory vitamin D analyses were performed independent of this study from day 0 to day 17 on ICU, explaining the observation period from day 0 to day 17. All stored leftover blood samples underwent the same analysis to quantify serum levels of 25(OH)D and 1,25(OH)2D at the Central Clinical Laboratory. Furthermore, markers of inflammation and disease severity, such as CRP, interleukin-6 (IL-6), TNF-alpha, lactate, leukocytes, procalcitonin, and fibrinogen, were assessed using established laboratory methods at the Central Clinical Laboratory, ensuring the accuracy and precision of the measurements.

### 2.4. Vitamin D Status

Holick et al. [26] defined vitamin D deficiency as a 25(OH)D concentration < 50 nmol/L, while vitamin D insufficiency is regarded as 52.5–72.5 nmol/L [26]. On the other hand, the Institute of Medicine mentioned in their Dietary Reference Intakes that most people are sufficient with serum 25(OH)D levels of at least 50 nmol/L [27]. A cutoff of lower than 25–30 nmol/L considerably increases the risk of osteomalacia and nutritional rickets and therefore is considered as being indicative of a severe vitamin D deficiency [28].

### 2.5. Statistical Analysis

We conducted three different types of analyses. Because all assessed variables were taken in the daily clinic routines, and due to clinical necessities, observations were unevenly distributed within time periods of ICU stay and across the 24 participants. In particular, toward later periods, less observations were available for the assessed variables. This led to uneven distributions of the assessed variables across and within participants. Therefore, we adopted an analysis strategy that was based on all available observations in the data set rather than on participants. As such, our analysis strategy did not take inter-individual variation into account, and, as we had *n* = 24 participants, all inferential analyses should be considered as exploratory and preliminary in nature. Additionally, we abstained from conducting detailed sub-group and confounder analyses, as the limited sample size precluded sufficient statistical power to discern subtle sub-group variations through the utilization of inferential statistics. Nonetheless, we provide detailed descriptions in Table 1. The focus of interpretation will lie in the descriptive analyses of the outcomes. Given that there is a lack of studies in this field and only a very limited number of available patients for observation, we think such an approach is warranted to generate and provide hypotheses for future research [29,30].

First, we conducted a descriptive analysis of vitamin D status in these critically ill patients. Further, we analyzed 25(OH)D and 1,25(OH)2D associations across the given time frames with values of CRP, fibrinogen, IL-6, TNF-alpha, procalcitonin, and leucocytes. To keep the analysis strategy consistent, we averaged (mean) inflammatory markers within participants and periods and submitted the resulting values to a Spearman rank-order correlation with 25(OH)D and 1,25(OH)2D. Additionally, we compared disease severity (PaO_2_/FiO_2_, SAPS III) with 25(OH)D and 1,25(OH)2D status on the day of admission (day 0, pre-ECMO). The literature assumes a correlation between severity and vitamin D. We calculated Spearman’s rank-order correlations to test for this suggested association based on all available data points. Lastly, the main analyses concerned time dependent changes in 25(OH)D and 1,25(OH)2D levels during ECMO therapy. Here, we considered three different time frames—an early period from day 0 to day 5, a middle period from day 6 to day 11, and a late period from day 12 to day 17 after admission. The grouping into these days was based on the availability of blood samples up to day 17 in the ICU (day of last collected blood sample). We averaged (mean) all observations per participant and periods (resulting in a range of 0 to 4 available values per period and participant). These values were then submitted to a non-parametric Kruskal–Wallis test in order to test for group differences between the three different time frames (early, middle, and late). In the case of significance differences, this was followed by between-group comparisons (Bonferroni–Holm-adjusted).

Due to the distribution characteristics of the observed variables, only non-parametric tests were performed. To allow for a deeper inspection of raw as well as distribution measures, all statistical analyses were converted into figures showing these aspects (raw values, boxplots, and violin plots). Calculations were performed using R statistics software (version 4.0.5, The R Foundation for Statistical Computing, Vienna, Austria).

## 3. Results

### 3.1. Descriptive Analysis

Within this retrospective study, 24 patients admitted to the ICU and treated with ECMO for COVID-19-related severe ARDS (*n* = 19; 79%) and non-COVID-19-related ARDS were analyzed. The mean age was 59 (SD ± 7) years. A total of 19 patients (79%) were male, the mean pre-ECMO SAPS III at ICU admission was 63 (SD ± 12), and the mean BMI was 29.2 (SD ± 6) kg/m^2^. Eleven (46%) patients died during the ICU stay. Half of the patients suffered from arterial hypertension and 6 patients (25%) had diabetes mellitus. The mean ICU length of stay (LOS) was 36 (SD ± 29) days. Demographic data and detailed baseline information are shown in Table 1.

According to Holick et al. [26], hypovitaminosis D (25(OH)D < 50 nmol/L (at least one measurement per patient below this range)) was detected in 18 (75%) of our patients, while 5 patients (21%) reported values of 25(OH)D lower than 30 nmol/L, indicating severe deficiency. Fifteen patients (62%) received vitamin D (Oleovit D3, Cholecalciferol; 400 IU/gtt.) substitution during their ICU stay. The vitamin D substitution dosage was 30–40 gtt. (12,000–16,000 IU) per week in 11 (46%) patients. One patient (4%) received 10 gtt. (4000 IU) per 48 h and 2 patients (8%) received 2 gtt. (800 IU) sublingual daily. One patient (4%) received 126 gtt. (50,400 IU) for the first four days within an ICU LOS of 28 days.

### 3.2. Vitamin D and Severity of Disease

**Figure 1:**
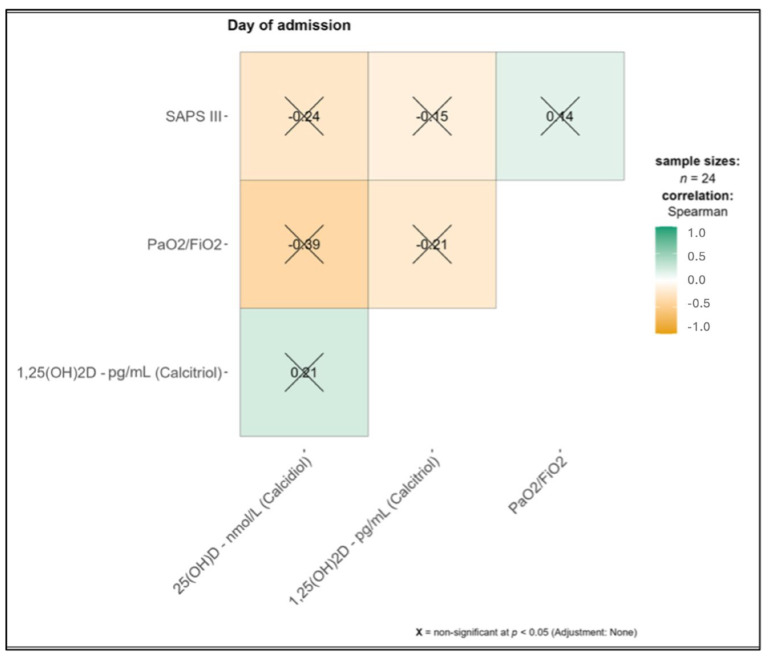
The severity of critical illness (SAPS III and PaO_2_/FiO_2_) was compared to levels of 25(OH)D and 1,25(OH)2D in 24 patients under ECMO therapy on the day of ICU admission. Correlations were analyzed by Spearman’s correlation and no significance was calculated. 1,25(OH)D = 1,25-dihydroxyvitamin D; 25(OH)D = 25-hydroxyvitamin D; SAPS III = Simplified Acute Physiology Score III.

On the day of ICU admission, levels of 25(OH)D and 1,25(OH)2D did not significantly correlate with the severity of illness expressed by the SAPS III and PaO_2_/FiO_2_ range. Details are shown in Figure 1.

### 3.3. Vitamin D and Inflammation

Our analysis of 25(OH)D, 1,25(OH)2D, and inflammatory markers (lactate, leukocytes, procalcitonin, TNF-alpha, IL-6, fibrinogen, and CRP) showed few significant correlations. TNF-alpha values were extremely high and exhibited only a slight drop in the period under review. IL-6 increased during the first 11 days of ICU treatment and dropped slightly again till day 17. Procalcitonin values were highest during the first five days of ICU treatment. All other inflammatory markers showed no particular outliers. The inflammatory markers within the ICU day groups are shown within Table 2.

**Figure 2:**
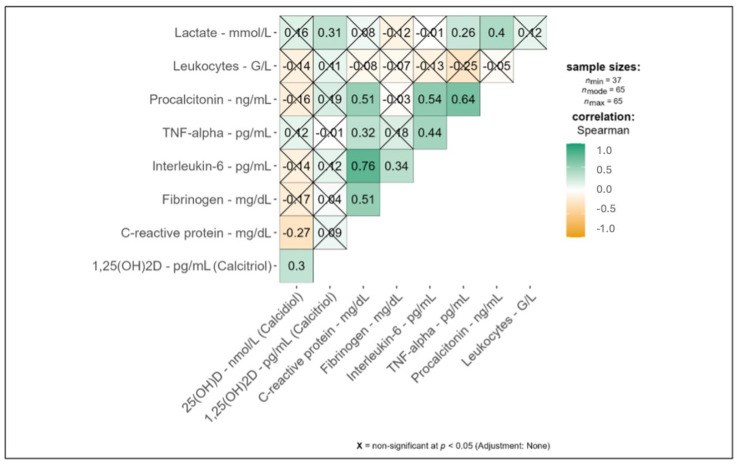
Correlations of inflammatory markers (lactate, leukocytes, procalcitonin, TNF-alpha, interleukin-6, fibrinogen, and C-reactive protein) with 25(OH)D and 1,25(OH)2D levels across the observation period (day 0 (= pre-ECMO) till day 17 (day 1 till day 17 = on ECMO)). Green indicates a positive correlation, while yellow indicates a negative correlation according to Spearman’s test. Here, 25(OH)D was significantly associated with 1,25(OH)2D (Spearman’s correlation *r* = 0.3 (*p* = 0.04; see Supplemental Table S1)), 1,25(OH)2D showed a significant positive association with lactate (Spearman’s correlation *r* = 0.31 (*p* = 0.03; see Supplemental Table S1)), and 25(OH)D showed a significant negative correlation with C-reactive protein (Spearman’s correlation *r* = −0.27 (*p* = 0.04; see Supplemental Table S1)). 25(OH)D = 25-hydroxyvitamin D; 1,25(OH)2D = 1,25-dihydroxyvitamin D; CRP = C-reactive protein; IL-6 = interleukin-6.

During the first 17 days in the ICU, 25(OH)D was significantly associated with 1,25(OH)2D (*p* = 0.04; Supplemental Table S1). Additionally, 25(OH)D showed a significant negative correlation with CRP (*p* = 0.04; r = −0.3; see Supplemental Table S1 and Figure 2), and, interestingly, 1,25(OH)2D showed a significant positive association with lactate (*p* = 0.03; r = 0.31; see Supplemental Table S1 and Figure 2). Also, 25(OH)D showed mean levels of 40.0 (±16.9) nmol/L in the early time frame, a mean of 44.1 (±18.3) nmol/L in the middle time frame, and a mean of 49.5 (±22.0) nmol/L in the late time frame. Finally, the values of 25(OH)D indicated hypovitaminosis D (<50 nmol/L) during all time frames. All correlations with inflammatory markers are shown in Figure 2.

### 3.4. Temporal Changes in Vitamin D Levels During ICU Stay

**Figure 3:**
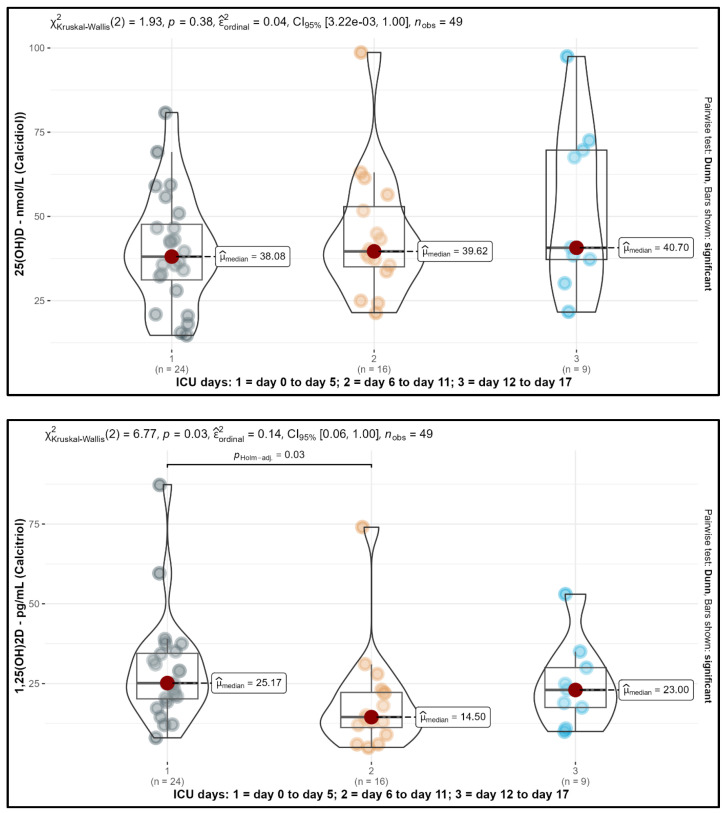
Comparison of ICU day group levels of 25(OH)D (upper figure) and 1,25(OH)2D (lower figure). Raw data (filled points), dispersion plots shown via a boxplot, median (red), and violin plots are displayed. Here, 1,25(OH)2D showed a significant decrease from the early (ICU day 0 to day 5) to the middle time frame (ICU day 6 to day 11) (*p* = 0.03; Bonferroni–Holm-adjusted), while 25(OH)D did not show significant changes. The vast majority of 25(OH)D values were below 75 nmol/L, indicating suboptimal or deficient status of 25(OH)D.

Levels of 25(OH)D and 1,25(OH)2D for the three ICU day groups are shown in Figure 3. Notably, 1,25(OH)2D showed a significant decrease from a median level of 25.17 pg/mL to 14.50 pg/mL from the early (ICU day 0 to 5) to the middle time frame (ICU day 6 to 11) (*p* = 0.03; Bonferroni–Holm-adjusted). For the late time frame (day 12 to 17), a non-significant trend of increasing values of 1,25(OH)2D could be observed up to 23.00 pg/mL. For all ICU day groups, mean 1,25(OH)2D levels were in the lower normal range.

Additionally, 25(OH)D did not show significant changes within all time frames; the median level at the early time frame was 38.08 nmol/L, increasing further in the middle and late time frames from 39.62 nmol/L to 40.70 nmol/L, indicating low values of 25(OH)D. A total of 96% of the 25(OH)D values were below 75 nmol/L, 75% were in the range of 30 to 75 nmol/L, and 21% were below 30 nmol/L. This indicated suboptimal or deficient status of 25(OH)D for the vast majority of our observations.

There were no significant differences in the results between vitamin D-supplemented and non-supplemented patients.

## 4. Discussion

Vitamin D exerts multiple effects on the immune system, affecting several types of immune cells. This might result in the decreased production of inflammatory cytokines, such as IL-6 and TNF-alpha [31], as well as the increased secretion of anti-inflammatory cytokines, such as IL-10 [32,33]. One mechanism by which vitamin D might reduce inflammation may derive from an overall switch from the more-inflammatory T-helper 1 (Th1)/Th17 response to the less-inflammatory Th2/Treg profile [14]. In turn, the resulting decreased production of proinflammatory markers could lead to reduced CRP synthesis. Another mechanism might be the inhibitory role of vitamin D in the regulation of NF-κB activation [34], which is an important route for the inflammatory response.

In our study, we found a significant negative correlation of 25(OH)D with CRP, which is a primary marker of inflammation. An inverse association between 25(OH)D and CRP has been shown in some [35] but not all [36] cross-sectional studies. For example, in 93 patients with COVID-19-related pneumonia, Saponaro et al. [37] found a similar inverse correlation between 25(OH)D and CRP (r = −0.21; *p* = 0.04), as in our study; however, no significance was observed in the crude correlation of 25(OH)D with TNF-alpha. However, since our data were only associative, and significance was only shown for CRP and no other established markers, like IL-6 or TNF alpha, we cannot definitively determine whether vitamin D plays a causal role in modulating inflammation.

CRP is known to be involved in the pathogenesis of COVID-19 and, for example, has been demonstrated to correlate with lung lesion appearance and with the severity of the disease [38], as well as potentially predicting the poor outcome of COVID-19 [39].

Several studies reported an association of vitamin D deficiency with worse outcomes in critically ill patients [33], including people suffering from severe COVID-19 [5,33,40]. However, we did not find any such association. In addition, other reports were not able to demonstrate an association between vitamin D deficiency and poor ICU outcomes [41]. For example, in a recent study investigating septic patients from Greece [42], no correlation was found between 25(OH)D levels and inflammatory biomarkers or the severity scores, with all patients showing hypovitaminosis D (25(OH)D < 50 nmol/L). Other studies also presented an increased prevalence of hypovitaminosis D in critically ill ICU patients, with prevalence rates between 40% and 70% [23]. In our study, 75% of the patients showed hypovitaminosis D.

A few papers have studied vitamin D kinetics in critically ill patients. For example, in 20 ICU patients from Poland, 25(OH)D levels decreased until the fourth measurement and then more or less stabilized over four to eight 12 h measurements [21]. In another large Austrian sample in critically ill patients from a vitamin D intervention trial, 25(OH)D levels remained stable and low in the placebo group but increased in the intervention group [20]. Additionally, no significant differences in 25(OH)D or 1,25(OH)2D levels were detected between days 1, 3, and 7 in ICU patients from Australia [22].

In our study, we found serial changes in 1,25(OH)2D in patients with severe COVID-19-related ARDS treated with invasive respiratory support and ECMO therapy within 17 days on ICU. In summary, although 25(OH)D levels were within normal ranges during the observational period, a significant decrease in 1,25(OH)2D from a median value of 25.17 pg/mL to 14.50 pg/mL between ECMO start and day 11 on ECMO could be demonstrated (*p* = 0.03).

The rate of the 1-alpha-hydroxylation of 25(OH)D by 1-alpha-hydroxylase (CYP27B1), primarily in the kidneys (but also partly in extrarenal cells like macrophages [43]), normally determines the serum level of 1,25(OH)2D, with the hydroxylation step being tightly regulated and affected by several factors, including parathyroid hormone (PTH), fibroblast growth factor 23, calcium levels, or 1,25(OH)2D itself [43].

Therefore, the inadequate 1-alpha-hydroxylation of 25(OH)D, possibly induced by an inhibitory effect of proinflammatory cytokines like TNF-alpha [44], might have been a cause of the decline in 1,25(OH)2D levels during the ICU stay of the patients in our study. Our patients presented very high TNF-alpha levels, and it has been shown that TNF-alpha can interfere with functional 1,25(OH)2D [45].

In addition to a possible reduction in 1,25(OH)2D synthesis, another reason for decreased levels of 1,25(OH)2D in our patients may have been a stimulated degradation of 1,25(OH)2D. Notably, 1,25(OH)2D is catabolized by CYP24A1 [43], and the inactivation of 1,25(OH)2D plays an important role in the availability of active vitamin D. In this regard, previous studies have shown that proinflammatory mediators can modify the expression of this enzyme in different cells [46,47]. In particular, TNF-alpha, but also IL-1β and IL-6, induced the expression of CYP24A1 in cultured human trophoblasts [46] and a combination of TNF-alpha/IL-1b in cultured airway epithelial cells [47].

### Limitations

Considering this retrospective, single-center project and the high adherence to current treatment guidelines including vitamin D substitution, this study was restricted to a small sample size, which only allowed for a descriptive and exploratory analysis. Therefore, one should be cautious regarding the overgeneralization of the results. However, according to our restrictive inclusion and exclusion criteria, we were able to observe unbiased total 25(OH)D and 1,25(OH)2D values of the same patients on multiple occasions, although only blood samples from leftover routine blood draws were collected. Regarding the duration of critical illness, there was no information regarding initial ICU admission, since most patients were transferred from other ICUs to receive ECMO support at our center. Thus, we considered previously reported pre-ECMO IMV values as the time frame of critical illness, according to which most patients were in the chronic phase of critical illness. This fact might have lead to a further limitation, as the patients probably already had a higher degree of inflammation upon admission to the ICU; therefore, the initial value of 25(OH)D could be correspondingly lower compared to the status before the onset of critical illness. Finally, potential cofounders such as nutritional support (parenteral nutrition) during ICU stays and corticosteroid therapy pre-ECMO were not included within this study due to its retrospective nature.

## 5. Conclusions

In conclusion, our study showed a decline in the active form of vitamin D which could have been due to several mechanisms, including the adverse effects of proinflammatory cytokines on vitamin D metabolism. Moreover, a direct comparison between groups is beyond the scope of this descriptive analysis but should be separately undertaken in future studies. All in all, our findings lead to the assumption that vitamin D plays an important role in critically ill patients, with many of them suffering from vitamin D deficiency. As pointed out in a recent review, vitamin D normalization of deficiency states could translate into positive health effects on multiple organ systems [48]. Vitamin D supplementation is a safe and simple intervention which should therefore be considered in this patient population.

However, further large-scale prospective studies controlling for different potential confounders are necessary to confirm our results and to investigate mechanisms and concrete test supplementation strategies in randomized trials. Routine vitamin D monitoring and substitution in critically ill patients, especially if patients are suffering from ARDS treated with ECMO, should be carried out to prevent hypovitaminosis D.

## Figures and Tables

**Table 1:** Baseline characteristics, ICU data, vitamin D substitution, comorbidities, and number of complications during the observation period.

	*n* = 24
Age, years (SD)	59 (±7)
Sex, male, no. (%)	19 (79%)
COVID-19-positive, no. (%)	19 (79%)
BMI, kg/m^2^, (SD)	29.2 (±6)
ICU length of stay, days (SD)	36 (±29)
SAPS III score, (SD)	63 (±12)
Pre-ECMO IMV duration, days (SD)	6 (±5.5)
IMV duration, days (SD)	21 (±8)
PaO_2_/FiO_2_ pre-ECMO (SD)	99 (±74)
Ventilator-free days till day 28 on ICU	4.1 (±6)
Death at ICU	11 (46%)
ECMO duration, days	19 (±14)
Vitamin D substitution (Oleovit D3, Cholecalciferol; 400 IU/gtt.)	
12,000–16,000 IU per week	11 (46%)
4000 IU per 48 h	1 (4%)
800 IU daily	2 (8%)
50,400 IU daily	1 (4%)
No substitution	9 (38%)
Comorbidities	
Arterial hypertension, no. (%)	12 (50%)
Chronic heart disease, no. (%)	4 (17%)
Obesity, no. (%)	5 (21%)
Diabetes mellitus, no. (%)	6 (25%)
Chronic respiratory disease, no. (%)	3 (13%)
Chronic kidney disease, no. (%)	0 (0%)
Cause of death	*n* = 11
Sepsis, no. (%)	1 (4%)
Multiorgan failure, no. (%)	6 (25%)
Intracerebral bleeding, no. (%)	2 (8%)
COVID-19 ARDS	2 (8%)

All qualitative parameters are given as count (percentage), and all quantitative parameters are given as mean (±SD). COVID-19 = coronavirus disease 2019; BMI = body mass index; ICU = intensive care unit; SAPS III = Simplified Acute Physiology Score III; ECMO = extracorporeal membrane oxygenation; IMV = invasive mechanical ventilation.

**Table 2:** Laboratory values of the ICU day groups.

	Early; Day 0–5 (Mean, SD)	Middle; Day 6–11 (Mean, SD)	Late; Day 12–17 (Mean, SD)
1,25(OH)2D, pg/mL	29.0 (±16.7)	20.9 (±17)	24.8 (±13.4)
25(OH)D, nmol/L	40.0 (±16.9)	44.1 (±18.3)	49.5 (±22)
CRP, mg/dL	15.6 (±6.7)	11.8 (±7.2)	13.3 (±7.5)
Fibrinogen, mg/dL	561.9 (±187.1)	509 (±175.3)	455.7 (±125.8)
IL-6, pg/mL	129 (±85.4)	312 (±796.4)	218.9 (±293.4)
TNF-alpha, pg/mL	8158.7 (±4191.7)	6393.7 (±2526.9)	6665.6 (±4018)
Procalcitonin, ng/mL	3.2 (±9.2)	1.1 (±2.8)	1.1 (±1.6)
Leukocytes, G/L	11.3 (±4.6)	10.3 (±3.9)	9.6 (±2.7)
Lactate, mmol/L	1.3 (±0.6)	1.2 (±0.8)	1.1 (±0.3)

All quantitative parameters are given as mean (±SD). 25(OH)D = 25-hydroxyvitamin D; 1,25(OH)2D = 1,25-dihydroxyvitamin D; CRP = C-reactive protein; IL-6 = interleukin-6; Day Group 1 = Day 0–5 on ICU; Day Group 2 = Day 6–11 on ICU; Day Group 3 = Day 12–17 on ICU.

## Data Availability

The data set supporting the conclusions of this article is available and saved at the Department of Anaesthesia, Intensive Care Medicine and Pain Medicine. Data are available from the authors upon reasonable request.

## Supplementary Materials

The following supporting information can be downloaded at https://www.mdpi.com/article/10.3390/medicina61050901/s1: Table S1: Correlations of inflammatory markers across the observation period (day 0 to day 17).

## Abbreviations

The following abbreviations are used in this manuscript:

1,25(OH)2D	1,25-dihydroxyvitamin D
25(OH)D	25-hydroxyvitamin D
ARDS	Acute respiratory distress syndrome
BMI	Body mass index
CRP	C-reactive protein
COVID-19	Coronavirus disease 2019
ECMO	Extracorporeal membrane oxygenation
ICU	Intensive care unit
IL-6	Interleukin-6
IMV	Invasive mechanical ventilation
LOS	Length of stay
PTH	Parathyroid hormone
SAPS	Simplified Acute Physiology Score III
