# Supplementary material for: Serial Changes in Vitamin D Status in Patients During Severe Acute Respiratory Distress Syndrome and Extracorporeal Membrane Oxygenation
Source: Medicina (Kaunas). 2025 May 16;61(5):901. doi: 10.3390/medicina61050901 (PMC12113077; doi:10.3390/medicina61050901)
Supplement: Supplementary file 1 [file medicina-61-00901-s001.zip › medicina-3596304-SI.pdf]

Supplemental Table S1: Correlations of inflammatory markers across the observation period (day 0 to day 17)

Variable 1	Variable 2	P value
**25(OH)D - nmol/L (Calcidiol)**	**1,25(OH)2D - pg/mL (Calcitriol)**	**0,04**
**25(OH)D - nmol/L (Calcidiol)**	**C-reactive protein - mg/dL**	**0,04**
1,25(OH)2D - pg/mL (Calcitriol)	C-reactive protein - mg/dL	0,55
25(OH)D - nmol/L (Calcidiol)	Fibrinogen - mg/dL	0,20
1,25(OH)2D - pg/mL (Calcitriol)	Fibrinogen - mg/dL	0,78
C-reactive protein - mg/dL	Fibrinogen - mg/dL	< 0,001
25(OH)D - nmol/L (Calcidiol)	Interleukin-6 - pg/mL	0,36
1,25(OH)2D - pg/mL (Calcitriol)	Interleukin-6 - pg/mL	0,46
C-reactive protein - mg/dL	Interleukin-6 - pg/mL	0,00
Fibrinogen - mg/dL	Interleukin-6 - pg/mL	0,02
25(OH)D - nmol/L (Calcidiol)	TNF-alpha - pg/mL	0,41
1,25(OH)2D - pg/mL (Calcitriol)	TNF-alpha - pg/mL	0,92
C-reactive protein - mg/dL	TNF-alpha - pg/mL	0,01
Fibrinogen - mg/dL	TNF-alpha - pg/mL	0,18
Interleukin-6 - pg/mL	TNF-alpha - pg/mL	< 0,001
25(OH)D - nmol/L (Calcidiol)	Procalcitonin - ng/mL	0,32
1,25(OH)2D - pg/mL (Calcitriol)	Procalcitonin - ng/mL	0,26
C-reactive protein - mg/dL	Procalcitonin - ng/mL	< 0,001
Fibrinogen - mg/dL	Procalcitonin - ng/mL	0,82
Interleukin-6 - pg/mL	Procalcitonin - ng/mL	< 0,001
TNF-alpha - pg/mL	Procalcitonin - ng/mL	< 0,001
25(OH)D - nmol/L (Calcidiol)	Leukocytes - G/L	0,31
1,25(OH)2D - pg/mL (Calcitriol)	Leukocytes - G/L	0,43
C-reactive protein - mg/dL	Leukocytes - G/L	0,53
Fibrinogen - mg/dL	Leukocytes - G/L	0,60
Interleukin-6 - pg/mL	Leukocytes - G/L	0,37
TNF-alpha - pg/mL	Leukocytes - G/L	0,06
Procalcitonin - ng/mL	Leukocytes - G/L	0,72
25(OH)D - nmol/L (Calcidiol)	Lactate - mmol/L	0,24
**1,25(OH)2D - pg/mL (Calcitriol)**	**Lactate - mmol/L**	**0,03**
C-reactive protein - mg/dL	Lactate - mmol/L	0,54
Fibrinogen - mg/dL	Lactate - mmol/L	0,34
Interleukin-6 - pg/mL	Lactate - mmol/L	0,92
TNF-alpha - pg/mL	Lactate - mmol/L	0,05

Procalcitonin - ng/mL	Lactate - mmol/L	0,01
Leukocytes - G/L	Lactate - mmol/L	0,34
